# Receptor for advanced glycation endproducts (RAGE) deficiency protects against MPTP toxicity

**DOI:** 10.1016/j.neurobiolaging.2011.12.006

**Published:** 2012-10

**Authors:** Peter Teismann, Kinnari Sathe, Angelika Bierhaus, Lin Leng, Heather L. Martin, Richard Bucala, Bernd Weigle, Peter P. Nawroth, Jörg B. Schulz

**Affiliations:** a Department of Neurodegeneration and Restorative Research, Center of Molecular Physiology of the Brain (CMPB) and Center of Neurological Medicine, University of Göttingen, Göttingen, Germany; b School of Medical Sciences, Institute of Medical Sciences, University of Aberdeen, Aberdeen, Scotland; c Department of Medicine, Clinical Chemistry, University of Heidelberg, Heidelberg, Germany; d Department of Medicine, Pathology, Yale University School of Medicine, New Haven, Connecticut, USA; e Eucodis, GmbH, Vienna, Austria; f RWTH University Hospital, Department of Neurology, JARA-BRAIN, Aachen, Germany

**Keywords:** MPTP, NF-κB, Parkinson's disease, RAGE, Neuroprotection, Microglia, Astrocytes

## Abstract

Parkinson's disease (PD) is a common neurodegenerative disorder of unknown pathogenesis characterized by the loss of nigrostriatal dopaminergic neurons. Oxidative stress, microglial activation and inflammatory responses seem to contribute to the pathogenesis. The receptor for advanced glycation endproducts (RAGE) is a multiligand receptor of the immunoglobulin superfamily of cell surface molecules. The formation of advanced glycation end products (AGEs), the first ligand of RAGE identified, requires a complex series of reactions including nonenzymatic glycation and free radical reactions involving superoxide-radicals and hydrogen peroxide. Binding of RAGE ligands results in activation of nuclear factor-kappaB (NF-κB). We show that RAGE ablation protected nigral dopaminergic neurons against cell death induced by the neurotoxin MPTP that mimics most features of PD. In RAGE-deficient mice the translocation of the NF-κB subunit p65 to the nucleus, in dopaminergic neurons and glial cells was inhibited suggesting that RAGE involves the activation of NF-κB. The mRNA level of S100, one of the ligands of RAGE, was increased after MPTP treatment. The dopaminergic neurons treated with MPP^+^ and S100 protein showed increased levels of apoptotic cell death, which was attenuated in RAGE-deficient mice. Our results suggest that activation of RAGE contributes to MPTP/MPP^+^-induced death of dopaminergic neurons that may be mediated by NF-κB activation.

## 1 Introduction

Parkinson's disease (PD) is a common neurodegenerative disorder of mostly unknown etiology, with the exception of some genetically inherited cases. It is characterized by disabling motor abnormalities, such as tremor, muscle stiffness, paucity of voluntary movements, and postural instability. Its primary neuropathological correlate is the specific loss of the nigrostriatal dopaminergic neurons, whose cell bodies reside in the substantia nigra pars compacta (SNpc) and whose nerve terminals project to the striatum (Przedborski et al., 2002). The subsequent deficit in brain dopamine induces most of the clinical motor features that are characteristic for the disease (Fahn and Przedborski, 2000).

Reactive oxygen species along with inflammatory processes have been implicated in the pathogenesis of PD (reviewed in Teismann and Schulz, 2004). Different factors have been linked to the induction of inflammation and in PD pathogenesis, one of those being the nuclear-factor-kappaB (NF-κB) (Dehmer et al., 2004; Ghosh et al., 2007; Hunot et al., 1997; Hunot et al., 1999). Previously it has been shown that ligation of the receptor for advanced glycation endproducts (RAGE) leads to a sustained activation of NF-κB (Bierhaus et al., 2001). RAGE expression has been found to be increased in incidental Lewy Body disease and was linked to oxidative stress, one of the main factors implicated in PD (Dalfo et al., 2005). RAGE is a multiligand receptor of the immunoglobulin superfamily of cell surface molecules. RAGE was first identified by its binding to advanced glycation end products (AGEs) (Kislinger et al., 1999). AGEs are a product of a series of reactions, the initial one being nonenzymatic glycoxidation that is followed by mechanisms involving reactions with reactive oxygen species. Another factor responsible for RAGE activation is the S100/calgranulin family of proinflammatory cytokine-like mediators (Donato, 1999; Hofmann et al., 1999; Kerkhoff et al., 1998). Immunization of mice with methylated serum albumin led to an inflammatory response which was inhibited by anti RAGE F(ab)′_2_ and anti-S100 F(ab)′_2_ fragment (Hofmann et al., 1999). A reduction in NF-κB activation was found in samples treated with these RAGE blockers, consistent with a central role for RAGE in generation of this inflammatory response (Hofmann et al., 1999). RAGE has also been proposed to be a mediator for amyloid-β (Aβ) peptide neurotoxicity in Alzheimer's disease (Yan et al., 1996) as well as being responsible for pathogenic Aβ assembly (Schmidt et al., 2001). Pathogenic Aβ species are able to induce RAGE expression in neurons, microglia and affected cerebral vasculature. The Aβ binding to RAGE has been shown to induce oxidative stress, microglial activation and inflammatory responses. RAGE expression is usually dependent on the presence of its ligands (S100, AGEs, Aβ). As the expression of these ligands is usually quite low, only mature animals express RAGE, but in low concentrations. However, the described ligands trigger the expression of RAGE (Brett et al., 1993; Park et al., 1998; Ritthaler et al., 1995; Yan et al., 1996). An accumulation of AGEs has not only been observed in Lewy bodies of PD patients but also in incidental Lewy Body disease (Munch et al., 2000), which is considered as a presymptomatic state of PD. These data imply that AGEs/RAGE may play an important role in the early stages of the disease.

Insights into the pathogenesis of PD and potential effects can be obtained by the use of the MPTP model of PD. The MPTP is a by-product of the chemical synthesis of a meperidine analogue and can induce a parkinsonian syndrome in humans almost indistinguishable from PD (Langston et al., 1983). Using this model we investigated not only the effect of RAGE-ablation on MPTP-induced toxicity, but also whether NF-κB translocation is attenuated in MPTP-treated RAGE deficient mice.

## 2 Methods

### 2.1 RAGE^−/−^ mice

RAGE^−/−^ mice were originally constructed on the SVEV129xC57BL/6 (129/B6) background and backcrossed into C57BL/6 mice as previously described in detail (Bierhaus et al., 2004). Experiments were performed with RAGE^−/−^ mice and wild type (wt) littermates after 10 backcrosses. All mice were housed individually with a 12-hour/12-hour light/dark cycle with free access to food and water. Procedures in this study were approved by the Animal Care and Use Committees at the Regierungspräsidium Braunschweig, Germany and the Home Office, Dundee, UK.

### 2.2 Animals and treatments

Eight-week-old male RAGE deficient (RAGE^−/−^) mice or their wt littermates were treated with either MPTP hydrochloride (Sigma, Deisenhofen, Germany) or saline. MPTP was administered in 0.1 ml of saline at a dose of 30 mg/kg i.p. (free base) at 24 h intervals over 5 consecutive days. For each study, 4–10 mice were used per group and were killed at selected times ranging from 0 to 21 days after the last injection. For HPLC measurements and tyrosine hydroxylase (TH) immunohistochemistry, mice were killed 3 weeks after the last MPTP injection. Control mice received saline only. MPTP handling and safety measures were in accordance with published guidelines (Przedborski et al., 2001).

### 2.3 RNA extraction and RT-PCR

Total RNA was extracted from selected mouse brain regions as described (Teismann et al., 2003). The primer mouse sequences were as follows: RAGE 5′-CTGAATCAGTCAGAGGAAGC-3′ (forward) and 5′-GGAGAAGGAAGTGCCTCAAG-3′ (reverse); S100 5′-GCTGACCACATGCCCCTGTAG-3′ (forward) and 5′-CTGGCCATTCCCCTCCTCTGTC-3′ (reverse), and GAPDH can be found in (Teismann et al., 2003). All products were analyzed and quantified by real-time PCR (Taqman, Applied Biosystems, Foster City, CA, USA).

### 2.4 Anti-AGE and anti-RAGE monoclonal antibody

Preparation and characterization of the anti-AGE monoclonal and, polyclonal antibody and the anti-RAGE monoclonal antibody used in this study has been described previously (Makita et al., 1992; Srikrishna et al., 2002).

### 2.5 Immunoblots

Mouse and human brain protein extracts were prepared as described (Teismann et al., 2003). Primary antibodies used were as follows: RAGE (1:1000), AGE (1:1000) or β-actin (1:10,000; Sigma). A horseradish-peroxidase-conjugated secondary antibody (1:10,000-1:25,000; Amersham Pharmacia, Freiburg, Germany) and a chemiluminescent substrate (Chemiglow, Biozym, Hess. Oldendorf, Germany) were used for detection. Bands were quantified by using a FluorChem 8800 digital image system (Alpha Innotech, San Leandro, CA, USA).

### 2.6 RAGE, AGE, TH, p65, glial fibrillary acidic protein (GFAP), BSI-IB4 and Iba1 immunohistochemistry

**Fig. 1:**
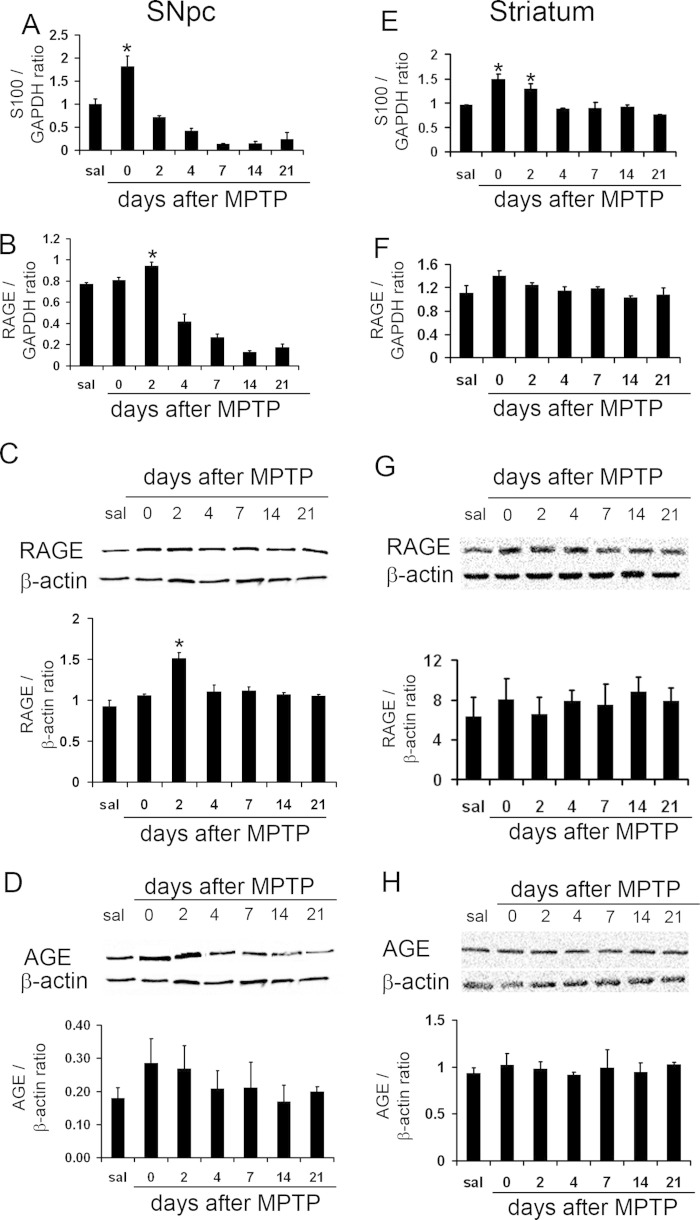
Ventral midbrain S100 and RAGE mRNA and RAGE and AGE protein expression after MPTP. S100 mRNA levels were increased by 0 days (A), RAGE mRNA levels by 2 days (B), after MPTP injection. RAGE protein contents were low in saline-injected mice (sal) (C) but were increased 2 days after MPTP treatment, whereas AGE expression remained unchanged (D). Striatal expression levels of RAGE and AGE remained unchanged (F–H), whereas S100B mRNA expression was significantly increased in the striatum at 0 and 2 days after MPTP (ANOVA followed Newman–Keuls post hoc test).

**Fig. 2:**
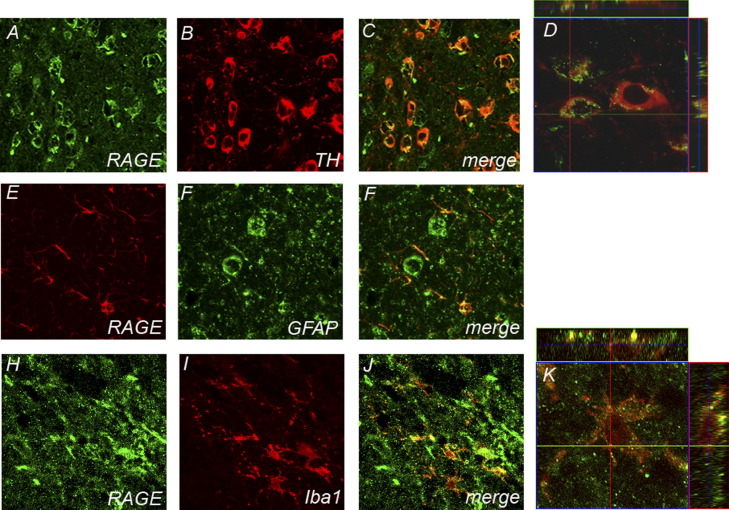
Ventral midbrain illustration of RAGE immunolocalization. RAGE-positive cells are abundant 2 d after MPTP, highly expressed in TH-positive neurons (red; A–C, 3D localization illustrated in D) and was also found in GFAP-positive cells (E–G; red) and Iba1-positive cells (red; H–J; 3D localization illustrated in K).

**Fig. 3:**
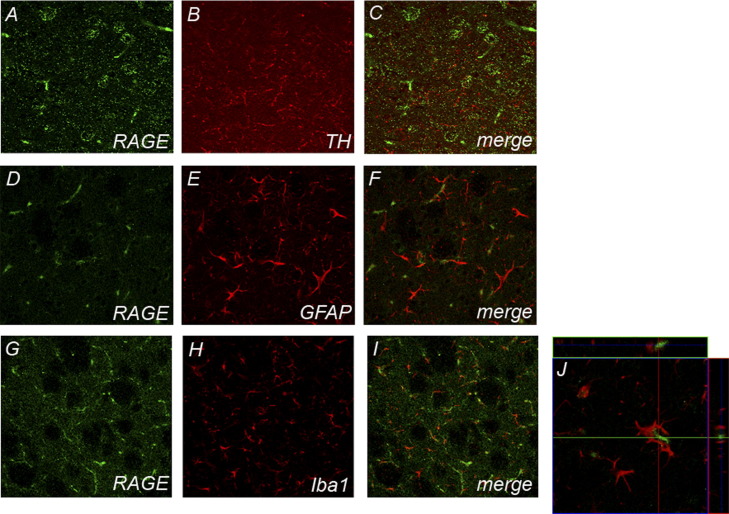
Striatal illustration of RAGE immunolocalization. Iba1-positive cells (red; G–I; 3D localization illustrated in J) show RAGE expression 2 d after MPTP. RAGE does not seem to be localized in TH-positive fibers (red; A–C) or GFAP-positive cells (D–F; red).

These were all performed according to our standard protocol for single or double immunostaining (Teismann et al., 2003). Primary antibodies were RAGE (1:100, Chemicon, Temecula, CA, USA), AGE (1:50), TH (1:1000; Chemicon, Temecula, CA, USA-polyclonal; DiaSorin, Stillwater, MN, USA-monoclonal), GFAP (1:100; Dako, Hamburg, Germany), p65 (1:50; Santa Cruz Biotechnology, Santa Cruz, CA, USA) and Iba1 (1:200; Wako Chemicals, Neuss, Germany). Activated microglia were detected by fluorescein isothiocyanate-labeled isolectin-B4 staining (BSI-IB4; 1:20; Sigma). Immunostaining was visualized by 3,3′-diaminobenzidine. RAGE and AGE staining were visualized by anti-mouse cy2 (1:300; Biotrend, Cologne, Germany). TH, GFAP and Iba1 staining were visualized by anti-rabbit cy3 (1:300) (Figs 2 and 3). To assess the colocalization of TH-positive neurons and p65, TH was visualized by anti-mouse cy2 (1:300), p65-staining was visualized by anti-rabbit cy3 (1:200) (Fig. 5). For assessment of increase of intracellular RAGE expression (see Fig. 2) sections were stained with mouse-anti-RAGE (1:1), visualized with Alexa Fluor 488 antimouse (1:300; Molecular Probes, Paisley, UK) and rabbit-TH (1:500) antibody, visualized with anti-rabbit cy3 (1:300) (Fig. 1). TH immunostaining was carried out on striatal and midbrain sections (Teismann et al., 2003). All stainings were examined by either regular light or confocal microscopy (LSM). The TH-stained and Nissl-stained SNpc neurons were counted by stereology using the optical fractionator method described (Teismann et al., 2003). The striatal density of TH immunoreactivity was determined as described (Teismann et al., 2003). The p65 translocation was assessed 2 days or 5 days after the first MPTP-injection as previously described (Dehmer et al., 2004). Sections were assessed as for stereological cell counts, but in this case for p65 localization. In all experiments the observer was “blind” as to the experimental condition of the animal.

### 2.7 Analysis of striatal dopamine levels and its metabolites

Mice were sacrificed 21 days after the last MPTP-injection and striatal levels of dopamine and its metabolites, dihydroxyphenylacetic acid (DOPAC) and homovanillic acid (HVA), were measured by high-performance liquid chromatography with electrochemical detection as previously described (Dehmer et al., 2004).

### 2.8 MPTP metabolism

Striatal MPP^+^ levels were determined by HPLC-UV detection (wavelength, 295 nm) (Teismann et al., 2003) 90 min after i.p. injection of 30 mg/kg MPTP.

### 2.9 Primary midbrain neuron cultures and immunocytochemistry

The mesencephalic floor plate was dissected from E12 RAGE^−/−^ mice and wt littermates and further processed for establishing dissociated cell cultures as previously described (Lingor et al., 2000). Cells were seeded on poly-L-ornithine/laminin (Sigma)-coated coverslips (10 mm diameter) at a density of 120,000 cells/cm^2^. Cultures were maintained at 37 °C in a humidified atmosphere and 5% CO_2_ in DMEM/F12 (Pan-Biotech, Aidenbach, Germany) plus the N1 supplements (Sigma) and antibiotics (Penicillin-Streptomycin-Neomycin (PSN) antibiotic Mixture (100X), Invitrogen, Karlsruhe, Germany) for 6 days. Media was changed 24 hours after establishing cultures, designated as day in vitro (DIV) 1, and subsequently every second day.

On DIV4, S100 (A and B, S6552, Sigma) (100 ng/mL), concentrations obtained from (Li et al., 2000), and/or MPP^+^ (2 μg/mL) were applied for 24 hours. On DIV6 cells were fixed with 4% paraformaldehyde (PFA) in PBS (pH 7.4). In order to identify dopaminergic neurons in cultures, immunocytochemical staining against TH was performed. To reduce unspecific binding, diluted goat serum (10% in PBS) was applied for 10 minutes at RT followed by incubation with a polyclonal anti-TH antibody (1:1000; Chemicon) and Hoechst 33258 (1:25,000; Molecular Probes, Karlsruhe, Germany). For fluorescence labeling a secondary Cy-3-labeled goat-anti-rabbit antibody (1:200; Biotrend) was used. Coverslips were mounted on glass slides and observed with a Zeiss Axioplan microscope.

TH-positive neurons and apoptotic nuclei were counted at 40-fold magnification in 6 different areas per well. In cell counts only TH-positive neurons possessing neurites were considered as such to avoid distortion by nonfunctional yet TH-positive cell debris. Cell survival is expressed as percentage standardized to the unchallenged and untreated control condition (100%). All experiments were performed at least in duplicate with at least 3 wells in parallel. All chemicals were freshly prepared and dissolved in cell culture medium immediately prior to application.

### 2.10 Statistical analysis

All values are expressed as the mean ± SEM. Differences among means were analyzed by using one-way or two-way ANOVA with time, treatment, or genotype as the independent factor. When ANOVA showed significant differences, pair-wise comparisons between means were tested by Newman–Keuls post hoc testing. In all analyses, the null hypothesis was rejected at the 0.05 level.

## 3 Results

### 3.1 MPTP induces RAGE and S100 expression in mouse ventral midbrain

To determine whether the expression of RAGE or its ligand S100 or AGE was affected during the nigrostriatal neurodegeneration, we assessed the contents of S100 mRNA, RAGE mRNA and RAGE and AGE protein in ventral midbrains (the brain region that contains the SNpc) and striatum of saline- and MPTP-injected mice, at different time points. Ventral midbrain S100, RAGE mRNA and protein were detected in saline-treated mice and were increased in MPTP-treated mice at day 0 and 2, respectively, after the last of the 5 MPTP injections (Fig. 1 A–C), whereas we found no increase in the expression of AGE (Fig. 1 D). In the striatum only S100 mRNA levels were significantly increased (Fig. 1 E), but not RAGE mRNA and protein as well as AGE protein levels (Fig. 1 F–H).

### 3.2 Induction of RAGE in SNpc dopaminergic neurons after MPTP administration

To elucidate the cellular origin of RAGE up-regulation in the ventral midbrain of MPTP-treated mice, we performed immunohistochemistry. Two days after the last injection, ventral midbrain RAGE immunostaining was detected to colocalize with TH staining in MPTP treated mice (Fig. 2 A–C). A 3D localization revealed that RAGE expression is punctuate and can be found at the cellular membrane (Fig. 2D). RAGE immunofluorescence did also colocalize with the microglial marker Iba1 (Fig. 2 E–G), and with the astrocytic marker GFAP (Fig. 2 H–J). In striatal staining RAGE expression was only detected in Iba1 positive cells (Fig. 3 G–J).

**Fig. 4:**
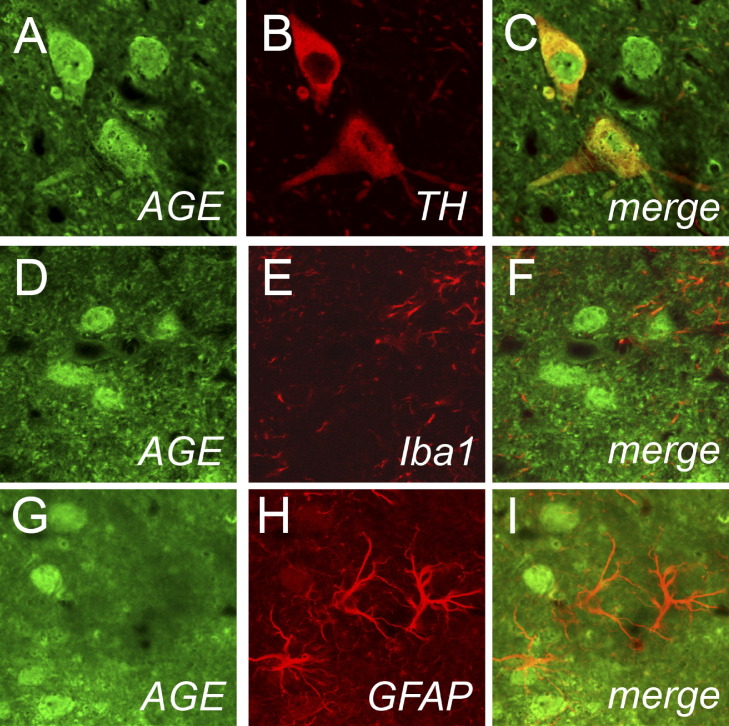
Ventral midbrain illustration of AGE immunolocalization. As RAGE-expression, AGE-positive cells are abundant 2 d after MPTP, highly expressed in TH-positive neurons (red; A–C) and not in Iba1-positive cells (D–F; red) or GFAP-positive cells (G–I; red).

We then investigated the cellular localization of AGE, a ligand for RAGE, after MPTP intoxication and found similar results regarding the cellular localization. AGE immunostaining was detected in dopaminergic neurons (Fig. 4 A–C), but AGE immunofluorescence did not colocalize with the microglial marker Iba1 (Fig. 4 D–F), or with the astrocytic marker GFAP (Fig. 4 G–I).

### 3.3 RAGE deficiency mitigates MPTP-induced neurodegeneration

**Fig. 5:**
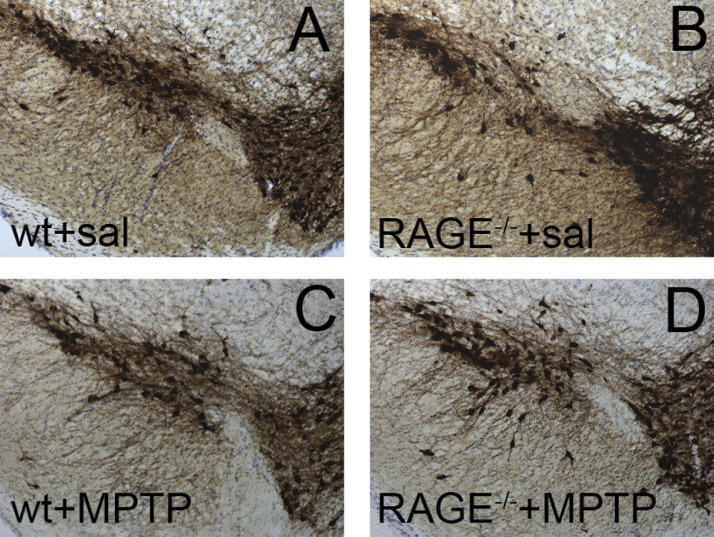
Effect of RAGE ablation on MPTP-induced neuronal loss. TH-positive cells of the SNpc appear comparable between saline-injected RAGE^−/−^ and RAGE^+/+^ mice (A and B). TH-positive neurons are more resistant to MPTP in RAGE^−/−^ (D) than in RAGE^+/+^ (C) mice. Cell counting was performed 21 d after MPTP. TH-positive neuronal counts are quantified in Table 1.

In light of the MPTP-induced up-regulation of RAGE and its ligands, we asked whether this receptor is involved in the nigrostriatal degeneration. Therefore, we compared the effects of MPTP in RAGE^−/−^ and RAGE^+/+^ mice. Stereological counts of SNpc dopaminergic neurons defined by TH and Nissl staining did not differ among the 2 genotypes after saline injections (Fig. 5, Table 1). SNpc dopaminergic neuron numbers were reduced in both genotypes after MPTP injections (Fig. 5 C and D, Table 1). However, in RAGE^−/−^ mice significantly more TH-stained SNpc neurons survived MPTP administration than in RAGE^+/+^ mice (Fig. 5, Table 1) (*p* = 0.035). MPTP-treated groups showed a high significant difference (*p* < 0.001) when compared vs. saline controls (*F* value = 33.438, degrees of freedom: between groups: 3, residual: 14, total: 17).

Loss of Nissl-positive cells confirmed, that the loss of TH-positive neurons corresponds to an actual loss of neurons (RAGE^+/+^ mice + saline = 14,520 ± 527, RAGE^−/−^ mice + saline = 15,450 ± 810, RAGE^+/+^ mice + MPTP = 7747 ± 375 and RAGE^−/−^ mice + MPTP = 9653 ± 465). In the striatum, the density of TH-positive fibers was decreased to 32.3% of saline values in MPTP-treated RAGE^+/+^ and to 35.6% in RAGE^−/−^ mice (Table 1). MPTP-treated groups showed a high significant difference (*p* < 0.001) when compared vs. saline controls (*F* value = 42.801, degrees of freedom: between groups: 3, residual: 14, total: 17). RAGE ablation also provided partial protection against the MPTP-induced loss of dopamine (*p* = 0.0499) and DOPAC (*p* = 0.021) but not against the loss of HVA in the striatum (Table 2).

MPTP-treated groups showed a high significant difference (*p* < 0.001) when compared vs. saline controls (dopamine: *F* value = 57.121, degrees of freedom: between groups: 3, residual: 13, total: 16; DOPAC: *F* value = 34.406, degrees of freedom: between groups: 3, residual: 12, total: 15; HVA: *F* value = 2.772, degrees of freedom: between groups: 3, residual: 9, total: 12).

**Fig. 6:**
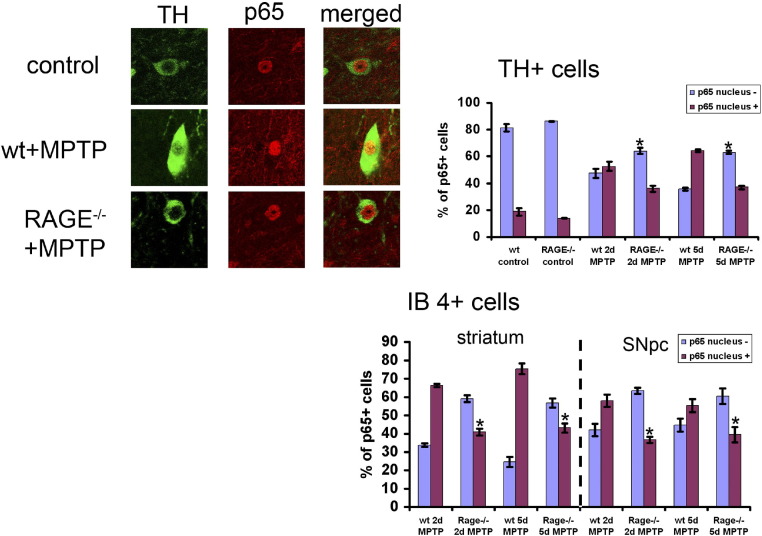
Effects of RAGE ablation on MPTP-induced NF-κB translocation in TH-positive neurons and glial cells. Costaining of TH-positive neurons with an antibody reactive against the p65/RelA subunit of NF-κB in the substantia nigra of animals that were left untreated (control), wild type animals treated with MPTP (MPTP), and RAGE^−/−^ mice treated with MPTP. Quantification (%) of TH-positive neurons and IB4-positive cells in the SNpc and IB4-positive cells in striatum that show translocation of p65/RelA staining from the cytosol to the nucleus after 2 and 5 doses of MPTP and block of this translocation in RAGE^−/−^ mice. *p < 0.05 compared with RAGE^+/+^ mice (n = 5) (ANOVA followed by Tukey's post-hoc test).

RAGE deficiency reduces NF-κB p65 translocation. Activation of NF-κB is a downstream signal of RAGE. We therefore investigated the translocation of the p65 subunit of NF-κB to the nucleus, which demonstrates the activation of NF-κB and is a prerequisite for its transcriptional activity. In controls, immunoreactivity for the NF-κB subunit p65/RelA was almost exclusively located in the cytosol, where it colocalized with TH (Fig. 6) and BSI-IB4 expression. In contrast, in MPTP-treated animals the p65/RelA immunoreactivity was translocated to the nucleus in TH-positive and IB4-positive cells. RAGE^−/−^ mice showed a reduced translocation of p65 to the nucleus in TH-positive and BSI-IB4-positive cells (Fig. 6).

### 3.4 RAGE deficiency mitigates microglia and astrocytic activation

As shown previously (Dehmer et al., 2004), BSI-IB4 immunostaining revealed a robust increase in the amount of activated microglia in the striatum and the SNpc after 2 and 5 doses of MPTP (Table 3). We also observed a marked increase of astrocytes in the striatum and SNpc (Table 3). RAGE^−/−^ mice showed reduced activation of microglia and less GFAP-positive cells in both striatum and SNpc after 2 and 5 injections of MPTP (Table 3).

### 3.5 RAGE deficiency does not impair MPTP metabolism in F10 generation

To ascertain that resistance to the neurotoxic effects of MPTP provided by RAGE deficiency was not due to alterations in MPTP metabolism, we measured concentration of MPP^+^ in striatal lysates 90 minutes after one injection of MPTP (30 mg/kg i.p.). MPTP metabolism was not significantly different in wt and RAGE^−/−^-mice. Striatal levels of MPP^+^ were not lower in MPTP-injected RAGE^−/−^ mice compared with RAGE^+/+^ mice (RAGE^−/−^ mice = 14.1 ± 0.98 and RAGE^+/+^ mice = 14.7 ± 0.84 μg/g wet tissue weight). Mice of this F10 generation were used for all experiments presented here.

### 3.6 RAGE deficiency protects against S100 and MPP^+^ induced toxicity

**Fig. 7:**
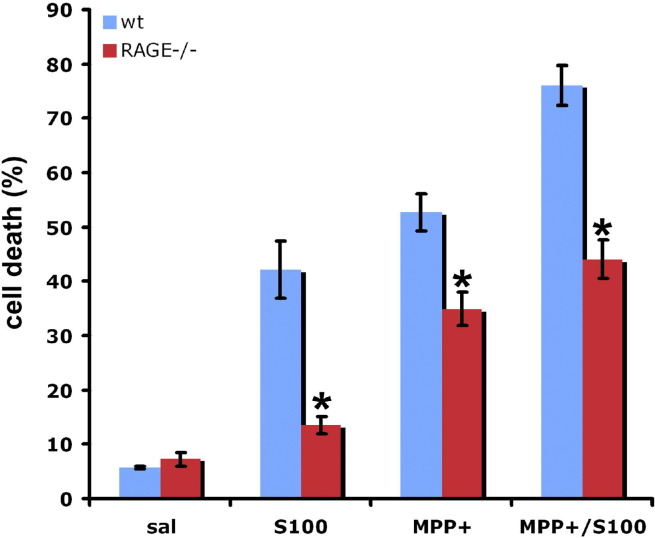
Treatment of primary dopaminergic neurons from RAGE^−/−^ mice with MPP^+^ resulted in a reduced number of cell showing apoptotic nuclei when compared with cells from wild type controls. MPP^+^ toxicity was enhanced, when primary dopaminergic neurons from RAGE^+/+^ mice where cotreated with S100, but not in cells from RAGE^−/−^ mice. *, p < 0.05, cells from RAGE^−/−^ mice compared with corresponding treatment of cells from RAGE^+/+^ mice (ANOVA followed by Newman–Keuls post hoc test).

To study the role of RAGE in S100- and MPP^+^ induced toxicity, primary dopaminergic neurons from RAGE^−/−^ mice and their wt littermates were exposed to S100 (100 ng/mL) and/or MPP^+^ (2 μM for 24 hours). Primary TH-positive neurons derived from RAGE-deficient mice were almost completely protected from S100 induced cell loss and partially protected from MPP^+^ induced toxicity. The combination of S100 and MPP^+^ induced more toxicity then either treatment alone in wild type animals. RAGE deficiency provided partial protection from combined S100/MPP^+^ toxicity (Fig. 7).

## 4 Discussion

Multiple factors have been implicated in the pathogenesis of PD and associated with MPTP-induced neurotoxicity. In this study we show an up-regulation of RAGE and microglia in the SNpc of mice treated with the neurotoxin MPTP. Significant alterations were not observed in the striatum, but this can be due to the fact that dopaminergic synapses only represent less than 15% of striatal structures (Pickel et al., 1981; Tennyson et al., 1974). RAGE expression was mainly present in TH-positive neurons and microglia, while its ligand AGE could mainly be detected in TH-positive neurons. Engagement of RAGE has been reported to magnify cell stress in different pathological settings (Stern et al., 2002). Rage-AGE interaction has been linked to the binding of amyloid fibers in Alzheimer's disease as well as propagating neurotoxic features of amyloid fibers (Yan et al., 1996). In addition, activation of RAGE has been linked to neuronal dysfunctions in diabetes, since activation of the RAGE-NF-κB axis contributes to the loss of pain perception in diabetic mice. Consistently, NF-κB activation and loss of pain perception are significantly reduced in streptozotocin-induced diabetic RAGE^−/−^-mice (Bierhaus et al., 2004).

In PD, a ∼70-fold increase of NF-κB activation in melanized midbrain neurons has been reported (Hunot et al., 1997). Furthermore, we have previously demonstrated that MPTP-administration leads to increased translocation of p65 to the nucleus in TH-positive neurons and glial cells (Dehmer et al., 2004). Since RAGE and RAGE-ligands accumulate in PD, the data led us to hypothesize that RAGE-dependent sustained NF-κB activation might contribute to the outcome of the disease. Consistently, the MPTP-mediated translocation of p65 was reduced in RAGE^−/−^-mice. RAGE^−/−^-mice treated with MPTP showed more surviving dopaminergic neurons than their control littermates. Also primary dopaminergic neurons from RAGE^−/−^-mice were partially protected against MPP^+^ induced cell death. These data implicate a role for RAGE mediated NF-κB translocation in MPTP-induced toxicity. The “classic” NF-κB-heterodimer consists of two proteins of 50 and 65 kDA, referred to as p50 and p65/RelA, respectively (for review see (Janssen and Sen, 1999; O'Neill and Kaltschmidt, 1997)). Surprisingly, ablation of the subunit p50 did not provide any beneficial effects in MPTP-induced toxicity (Teismann et al., 2001). As mice deficient in the p65/RelA subunit of NF-κB are embryonically lethal (Beg et al., 1995), no data exists on the function of the p65/RelA subunit of NF-κB MPTP toxicity. Very recently it has been shown that inhibition of NF-κB activation by the use of NF-κB essential modifier-binding domain peptides (NBDs) provides neuroprotection against MPTP-induced toxicity, by preventing the expression of proinflammatory molecules (Ghosh et al., 2007), thus demonstrating a role for NF-κB in the MPTP-model of PD. S100B could be one of the factors involved in NF-κB activation, as it can activate p65/c-Rel transcription in a RAGE dependent manner (Kogel et al., 2004). Moreover, NF-κB mediates neuronal nitric oxide synthase (nNOS) induction in a cellular model of MPTP-toxicity (Carbone et al., 2008). Furthermore, inhibition of NF-κB with 1,1-bis(3′-indolyl)-1-(*p-t*-butylphenyl)methane resulted in a reduction of caspase activity and nNOS activation (Carbone et al., 2009). Overall, a partially reduced NF-κB activation, but not total inhibition, could be an explanation for the lesser effect observed on loss of TH-positive neurons after MPTP.

Since RAGE expression was found in TH-positive dopaminergic neurons it was surprising that RAGE-deficiency did not extend its neuroprotective effect to striatal dopaminergic fibers. It has been reported that glial changes are more significant to MPTP-induced cell death in the SNpc than in the striatum (Liberatore et al., 1999) due to their proximity to dopaminergic structures and are more robust in the SNpc (Teismann and Schulz, 2004). In our study the protective profile mediated by RAGE deficiency did not extend to striatal dopaminergic fibers but also RAGE expression was not affected by MPTP significantly in the striatum (Fig. 1). The neurites seem to be more prone to toxic insults as different studies using MPTP-induced cell death indicate. In many former studies interfering with apoptotic or inflammatory pathways in MPTP-induced cell death resulted in protective effects on dopaminergic somata of the SNpc but not on striatal dopaminergic terminals (Dehmer et al., 2000; Liberatore et al., 1999; Tieu et al., 2004). Dopamine content was partially rescued in RAGE deficient animals, indicating that even though there was no significant change in numbers of dopaminergic terminals, functionality of the neurons was sustained.

The MPTP-protocol, which used (5 × 30 mg/kg i.p.) also leads to an inflammatory response contributing to the cell death process. Despite the fact that microglia can be beneficial reports using the MPTP-model describe a deleterious effect associated with the activation of microglia (Wu et al., 2003). In humans MPTP leads to a slowly progressive degeneration as demonstrated in a study of MPTP intoxicated patients, which revealed an active ongoing degenerative and inflammatory process in the SNpc 3–16 years after MPTP exposure (Langston et al., 1999).

The MPTP leads to a time-dependent increase in expression of S100B, another ligand of RAGE, localized in astrocytes (Fig. 2) (Himeda et al., 2006; Muramatsu et al., 2003). S100B, the predominant form of S100 in the brain is both neuroprotective and neurotoxic, depending on the dose (Huttunen et al., 2000). RAGE activation by nanomolar S100B leads to an increased expression of the antiapoptotic protein Bcl-2 whereas in micromolar concentration S100B induces apoptosis in an oxidant and MAP-kinase dependent manner (Huttunen et al., 2000).

Interestingly we found a large decrease after a significant rise in S100B and RAGE mRNA levels in the SNpc. We can only hypothesize that S100B might lead to self-inhibition through a feed-back loop after the initial increase. The same could also hold true for RAGE, but it might also be related to the loss of TH-positive neurons, as these numbers are significantly reduced. As low levels of S100B expression is associated with neuroprotection, the underlying mechanism could also be compensatory, to protect the remaining neurons due to lowered S100B expression. Nevertheless, these findings will need further investigation.

The authors (Himeda et al., 2006; Muramatsu et al., 2003) suggest, that GFAP-positive astrocytes, which secrete S100B, participate in MPTP-induced toxicity, and thus in the pathogenesis of PD. S100B is capable of activating COX-2, but this depends on the presence of RAGE (Bianchi et al., 2010).

We also detected a reduced number of microglia in MPTP-treated RAGE^−/−^ mice, indicating, that the inflammatory process was attenuated due to RAGE ablation. Preceding reports have linked microglia activation with MPTP-induced toxicity (Wu et al., 2002; Wu et al., 2003), which in turn has been linked to the formation of inducible nitric oxide synthase (iNOS) (Dehmer et al., 2000; Liberatore et al., 1999). Furthermore, we found a reduction in microglia showing p65 translocation, implying that iNOS expression might be reduced as described (Dehmer et al., 2004). This may lead to the increased number of surviving dopaminergic neurons seen in RAGE^−/−^ mice. Microglia activation seems to be secondary to neuronal damage, and thus RAGE ablation might not mediate a very pronounced neuroprotective effect.

As we found a rather weak effect of neuroprotection due to RAGE ablation, we hypothesize that this pathway is only marginally involved in the neurodegenerative process, especially as COX-2 ablation shows a more profound neuroprotective effect in a more severe model (Teismann et al., 2003). However, S100B was also shown to activate p38, ERK1/2 and JNK in Alzheimer's disease (Kim et al., 2004), of which S100B mediated JNK and ERK1/2 activation seems again be dependent on the presence of RAGE (Bianchi et al., 2010). As mice lacking JNK showed a more pronounced neuroprotective effect then we observed in RAGE^−/−^ mice (Hunot et al., 2004), it seems unlikely that RAGE activates the JNK pathway in the MPTP model.

Another factor, which could contribute to RAGE activation is its ligand high mobility group Box 1 (HMGB1). This protein is released from necrotic and inflammatory cells leading to an inflammatory response (Lotze and Tracey, 2005), features seen in PD and the MPTP-model. It has also been shown that in stroke inhibition of HMGB1 release as well as RAGE ablation leads to a neuroprotective effect (Muhammad et al., 2008). The data show, that RAGE expression on glial cells mediates HMGB1 effects (Muhammad et al., 2008), which could also be the case in the MPTP-model.

In a final set of experiments we tested the influence of the ligand S100 on survival of dopaminergic neurons. Addition of S100 to dopaminergic neurons led to increased cell death and also increased MPP^+^ mediated neurotoxicity. Using primary dopaminergic neurons from RAGE^−/−^ mice we were able to ablate the deleterious effect mediated by the addition of S100, although not to full extend. But RAGE is not the only mediator of S100, as S100 also activates a multitude of other factors, most importantly iNOS (Hu et al., 1996) and interleukin-1β (IL-1β) (Koppal et al., 2001). It is very likely, that part of the neurotoxic effect after MPP^+^ is mediated by these targets of S100B, which do not require RAGE.

Our results further support the evidence for inflammatory processes contributing to MPTP-induced cell death. They suggest a role for RAGE-mediated microglia response and subsequently RAGE-mediated cell death in MPTP-induced neurotoxicity. Treatment strategies aimed at inhibiting the inflammatory response in connection with blocking RAGE could prove beneficial in halting/slowing the progression of PD.

## Disclosure statement

None of the authors have any actual or potential financial conflicts or conflict of interest related to this study.

## Figures and Tables

**Table 1:** Effect of RAGE ablation on MPTP-toxicity

	SNpc: no. of TH-positive neurons		Striatum: TH-positive fibers, OD × 100	
	RAGE^+/+^	RAGE^−/−^	RAGE^+/+^	RAGE^−/−^
Saline	10200 ± 266	10253 ± 565	24.41 ± 0.75	24.71 ± 2.07
MPTP	4640 ± 103⁎	5472 ± 193⁎^,^†	7.89 ± 1.04⁎	8.80 ± 0.96⁎

Values are mean ± SEM for four to six mice per group. ANOVA followed by Newman–Keuls post hoc test.

⁎ *p* < 0.001 compared with the other groups of saline-treated mice.

† *p* = 0.035 compared with MPTP-injected RAGE^+/+^ mice.

**Table 2:** Striatal concentrations of catecholamines

	Dopamine	DOPAC	HVA
RAGE^+/+^ + saline	15.80 ± 0.42	3.13 ± 0.19	0.92 ± 0.02
RAGE^−/−^ + saline	15.34 ± 1.46	2.95 ± 0.16	1.04 ± 0.15
RAGE^+/+^ + MPTP	4.15 ± 0.64⁎	1.12 ± 0.12⁎	0.73 ± 0.02⁎
RAGE^−/−^ + MPTP	6.38 ± 0.25⁎^,^†	1.68 ± 0.08⁎^,^#	0.81 ± 0.04⁎

RAGE^−/−^mice are partially protected from MPTP-induced decrease of striatal dopamine and DOPAC concentrations at 21 days after the last MPTP injection, but are not protected against decrease of HVA. Values are mean ± SEM for 3 to 6 mice per group. ANOVA followed by Newman–Keuls post hoc test.

⁎ *p* < 0.001 compared with the other groups of saline-treated mice.

† *p* = 0.0499 compared with MPTP-injected RAGE^+/+^ mice.

# *p* = 0.021 compared with MPTP-injected RAGE^+/+^ mice.

**Table 3:** Numbers of IB 4 and GFAP positive cells in striatum and SNpc

	Striatum		SNpc	
	IB 4-positive	GFAP positive	IB 4-positive	GFAP positive
wt + saline		9.5 ± 3.3		20.7 ± 1.2
RAGE^−/−^ + saline		8.1 ± 0.6		18.2 ± 2.6
wt + MPTP 2x	86.1 ± 5.7	84.8 ± 15.9	98.5 ± 9.5	127 ± 14.8
RAGE^−/−^ + MPTP 2x	77.5 ± 2.9	46.8 ± 1.1⁎	89.3 ± 7.3	44.1 ± 5.6⁎
wt + MPTP 5x	79.0 ± 4.0	104.4 ± 7.5	103.0 ± 12.1	107.6 ± 11.8
RAGE^−/−^ + MPTP 5x	62.0 ± 4.0	66.0 ± 7.0⁎	87.5 ± 5.8⁎	63.7 ± 2.6⁎

Treatment with two (2x) or five (5x) doses of MPTP activated microglia (IB4-positive cells) and astrocytes (GFAP positive cells) in the striatum and SNpc. This activation was reduced in RAGE^−/−^ mice. Data are mean ± SEM, in number of IB 4 or GFAP-positive cells within an area of 0.25 mm^2^. In controls, no IB 4 positive cells were detected. ANOVA followed by Newman–Keuls post hoc test.

⁎ *p* < 0.05 compared with MPTP treated wild type mice.
